# Retraction: Determining engineering properties of ultra-high-performance fiber-reinforced geopolymer concrete modified with different waste materials

**DOI:** 10.1371/journal.pone.0346378

**Published:** 2026-04-06

**Authors:** The PLOS One Editors

The *PLOS One* Editors retract this article [1,2] due to concerns about reliance on retracted work cited in References 6, 12, 42, 43, 46, 49, 53, and 54.

RMG and JdPG agreed with the retraction. FA, OZ, SA, and MMA either did not respond directly or could not be reached.
